# Rocuronium bromide suppresses esophageal cancer via blocking the secretion of C–X–C motif chemokine ligand 12 from cancer associated fibroblasts

**DOI:** 10.1186/s12967-023-04081-y

**Published:** 2023-04-08

**Authors:** Jingyi Li, Xuefeng Gu, Guoqing Wan, Yuhan Wang, Kaijie Chen, Qi Chen, Changlian Lu

**Affiliations:** 1 Shanghai Key Laboratory of Molecular Imaging, Zhoupu Hospital, Shanghai University of Medicine & Health Sciences, No. 279, Zhouzhu Road, Shanghai, 201318 China; 2 Qiqihar Medical University, Qiqihar, 161006 Heilongjiang Province China

**Keywords:** Rocuronium bromide, Esophageal cancer, Cancer associated fibroblasts, CXCL12, Autophagy

## Abstract

**Background:**

Cancer associated fibroblasts (CAFs) communicate metabolically with tumor genesis and development. Rocuronium bromide (RB) is reported to exert certain inhibitory effect on tumor. Here, we investigate the role of RB in esophageal cancer (EC) malignant progression.

**Methods:**

Tumor xenograft models with EC cells were locally and systemically administrated with RB to detect the influence of different administrations on tumor progression. Mouse CAFs PDGFRα^+^/F4/80^−^ were sorted by Flow cytometry with specific antibodies. CAFs were treated with RB and co-cultured with EC cells. The proliferation, invasion and apoptosis assays of EC cells were performed to detect the influences of RB targeting CAFs on EC cell malignant progression. Human fibroblasts were employed to perform these detections to confirm RB indirect effect on EC cells. The gene expression changes of CAFs response to RB treatment were detected using RNA sequencing and verified by Western blot, immunohistochemistry and ELISA.

**Results:**

Tumors in xenograft mice were observed significantly inhibited by local RB administration, but not by systemic administration. Moreover EC cells did not show obvious change in viability when direct stimulated with RB in vitro. However, when CAFs treated with RB were co-cultured with EC cells, obvious suppressions were observed in EC cell malignancy, including proliferation, invasion and apoptosis. Human fibroblasts were employed to perform these assays and similar results were obtained. RNA sequencing data of human fibroblast treated with RB, and Western blot, immunohistochemistry and ELISA results all showed that CXCL12 expression was significantly diminished in vivo and in vitro by RB. EC cells direct treated with CXCL12 showed much higher malignancy. Moreover cell autophagy and PI3K/AKT/mTOR signaling pathway in CAFs were both suppressed by RB which can be reversed by Rapamycin pretreatment.

**Conclusions:**

Our data suggest that RB could repress PI3K/AKT/mTOR signaling pathway and autophagy to block the CXCL12 expression in CAFs, thereby weakening the CXCL12-mediated EC tumor progression. Our data provide a novel insight into the underlying mechanism of RB inhibiting EC, and emphasize the importance of tumor microenvironment (cytokines from CAFs) in modulating cancer malignant progression.

**Supplementary Information:**

The online version contains supplementary material available at 10.1186/s12967-023-04081-y.

## Introduction

Esophageal cancer (EC) is one of the malignant tumors with high morbidity and mortality worldwide. Although neoadjuvant chemoradiation therapy has been widely administered in the treatments of EC with locally advanced or lymph node positive tumors, and 19% of patients with pathologic complete response show 86% three-year overall survival and 80% recurrence-free survival rates [1], nearly 60% of patients fail to respond to neoadjuvant chemoradiotherapy reducing the success of surgery [2, 3]. EC patients are still suffering from the advanced stages and poor prognosis, which is required for improvements in the management and treatment of cancer [4].

In recent years, the connection between anesthetics and tumor from the perspective of perioperative medicine has attracted the raising attention [5–7]. Researchers have reported that sevoflurane and desflurane could stimulate malignancy of ovarian cancer, osteosarcoma, glioma and lung cancer [8, 9], while cisatracurium can suppress colorectal cancer progression [10]. However, in actual clinical practice, the total-body dosage and short-term treatment duration of anesthetic drugs are not suitable for tumor treatment.

In tumor microenvironment (TME), fibroblasts communicate with tumor cells through various mechanisms, such as lactic acid shuttle, alteration of glycolysis activity, enhancement of autophagy secretion, etc. [11–13]. In addition, fibroblasts provide metabolic links between fibroblasts and tumor cells through metabolic derivatives such as cytokines release, creating a favorable metabolic microenvironment for tumor progression. Epigenetic regulation, autophagy and cytokines secreted by tumor cells can influence these cancer associated fibroblasts (CAFs) [14, 15]. CAFs regulate tumor cell phenotype, metastasis and angiogenesis via releasing growth factors, pro-inflammatory cytokines and chemokines including C–X–C motif chemokine ligand 12 (CXCL12) [16, 17], which is robustly expressed in most cancers, and plays important roles in cancer growth, migration, invasion and angiogenesis [18–20].

Nonetheless, the regulatory relationship between anesthetics and CAFs is not clear. We suspect that whether anesthetics can exert a tumor repressive effect on EC through CAFs. Therefore, our project aims to study rocuronium bromide (RB) effect on EC cells in vitro and in vivo, and gene expression change of CAFs induced by RB treatment, and we mainly focus on the secretory protein CXCL12, and study the regulatory mechanism of CXCL12 expression by RB in CAFs. This study will figure out the therapeutic effect of RB on EC from a novel perspective, and the data can provide a resource to search more exocrine factors in tumor microenvironment for cancer treatment.

## Materials and methods

### Cell culture

Human EC TE-1 and ECA-109 cells, and Human fibroblast HS-27, TIG-1 and CRL-7815 cells, were cultured within DMEM and 1640, respectively, containing fetal bovine serum 10% and antibiotics (Thermo Fisher Scientific). Fibroblasts were pre-treated with multiple conditions, then passaged and co-cultured with EC cells using μ-Slide 2 Well System (Ibidi, Martinsried, Bavaria, Germany). The treatment conditions of drug cells are as followed: RB treated for 12 h with 10–320 μg/mL (PHR2397, Merck, Darmstadt, Hessian, Germany); human CXCL12 recombinant protein treated for 12 h with 10 μg/mL (RP-8658, Thermo Fisher Scientific); rapamycin (V900930, Merck, 500 nM for 8 h) [21, 22].

### Xenograft mouse study

Four-week-old female BALB/c nude mice (Shanghai Sippr BK Laboratory Animal, China) were used for xenograft tumor experiments. The transplantation and drug administration were carried out in strict accordance with the International Committee's Guide for the Care and Use of Laboratory Animals. For xenograft tumor experiment, 5 × 10^6^ TE-1 or ECA-109 cells mixed with matrix-gel were subcutaneously inoculated into the back of the right upper limb of nude mice (3 mice per group). For systematical administration, after 1 week subcutaneous inoculation, RB with different concentrations (0, 10 and 20 mg/kg) [23] were administrated with the nude mice every 2 days for additional 2 weeks. For local administration, the gradient concentrations of RB (0, 40 and 80 μg *per* mouse) were given in the connective tissue layer underneath the tumor every two days for additional 2 weeks. Then mice were executed with carbon dioxide asphyxia, xenograft tumors were measured and removed for histochemical analysis.

Immunohistochemistry (IHC) was used to detect the expression change of ATG5 and CXCL12 influenced by RB treatment in vivo, xenograft tumors and their adjacent tissues were fixed, embedded within paraffin, and sectioned routinely. Antibodies against ATG5 (No. #12994, CST, Beverly, MA, USA) at 1:500 dilution and CXCL12 (Cat No. #3530, CST) at 1:1000 dilution were used respectively.

### RNA sequencing

Total RNAs were isolated from 1 × 10^7^ fibroblasts, HS-27, TIG-1 and CRL-7815 cells, treated with and without RB treatment using TRIZOL (Thermo Fisher Scientific) [24]. CDNA library was constructed using the NEBNext Ultra Directional RNA Library Prep Kit for Illumina (NEB), and sequenced on an Illumina HiSeq platform. Raw data were screened to remove low-quality reads and adaptors using Trimmomatic, and aligned to human genome (GRCh38) using Hisat2. Ensembl Genome Browser database was used for transcript and gene annotation (http://www.ensembl.org/index.html). The R package ClusterProfiler was used to annotate the Differential expressed genes (DEGs) using Gene Ontology (GO) terms [25, 26]. Data was uploaded to ArrayExpress (E-MTAB-11728).

### Colony formation assay and invasion assay

For colony formation assay, 1 × 10^5^ cells were disseminated and cultured for 2 weeks, and then cross-linked in 1% paraformaldehyde followed by GIMSA staining for 15 min and colonies were counted. For invasion assay*,* 1 × 10^5^ cells were cultured in transwell coated with matrigel for 24 h (Corning, NY, USA). The cells at the lower chamber film were washed with PBS for 2 min, fixed with 1% paraformaldehyde and then stained with crystal violet followed by counting under microscope at 200× magnification.

### Flow cytometric assay

For murine CAFs isolation, tissues adjacent to xenograft tumor were scissored carefully followed by 1% trypsin digestion at 37 °C. After PBS washes 3 times, cells were respectively incubated with FITC anti-mouse PDGFRα (Cat. No. 130-109-735, Miltenyi Biotec, Bergisch Gladbach, Nordrhein-Westfalen, Germany) and PE anti-mouse F4/80 antibody (Cat. No. 130-116-499, Miltenyi Biotec). The cells were sorted by FACS Calibur FCM (BD Biosciences, Franklin Lakes, NJ, USA), PDGFRα^+^/F4/80^−^ cells were considered as CAFs as previously described [27, 28].

### Western blot

The protein lysate was analyzed by SDS-PAGE and transferred to PVDF membranes (Bio-Rad Laboratories, Hercules, CA, USA). The membrane was blocked with 5% fat-free milk in PBST for 30 min, followed by incubation overnight at 4 °C with final dilution of primary antibodies against CXCL12 (Cat No. #3530), IL8 (Cat No. #94407), TGF-β (Cat No. #3711), HGF (Cat No. #52445), mTOR (Cat No. #2983), AKT (Cat No. #4685), AMPK (Cat No. #5831), LC3II/I (Cat No. #4108), ATG5 (Cat No. #12994), p-mTOR (Cat No. #5536), p-AMPK (Cat No. #50081), p-AKT (Cat No. #4060) or GAPDH (Cat No. #5174) all from CST (Beverly, MA, USA). Antibodies on membranes could be stripped using stripping buffer (Cat No. ab270550, Abcam, Cambridge, MA, USA) with gently shaking at 52 °C for 30 min for other blotting examination. Protein bands hybridized with primary and secondary antibodies on membranes were detected using ultrasensitive ECL chemiluminescence reagent (Beyotime Biotechnology, Shanghai, China) and exposed to film. Band intensity was quantified as the mean ± SD of three independent experiments.

### Enzyme-linked immunosorbent assay (ELISA)

To examine cytokines secreted by fibroblasts treated with or without RB for 12 h, the method of *ELISA* was performed using antibodies for CXCL12 (E-EL-H0052c, Elabscience, Wuhan, China), IL8 (E-EL-H6008, Elabscience), HGF (E-EL-H0084c, Elabscience) and TGF-β (E-EL-H1587c, Elabscience).

### Statistical analysis

Data is showed as the mean ± standard deviation for at least three independent duplications. The variables between two groups were analyzed using Student’s *t*-test. All t-test data were performed Shapiro Wilk check for the normal distribution. Analyses of multiple groups’ comparisons were performed using one-way or two-way ANOVA followed by Dunnett’s multiple comparisons compared with control. The *P*-value less than 0.05 was considered as statistical significance.

## Results

### RB exerted an inhibitory effect on EC in vivo and in vitro

**Fig. 1:**
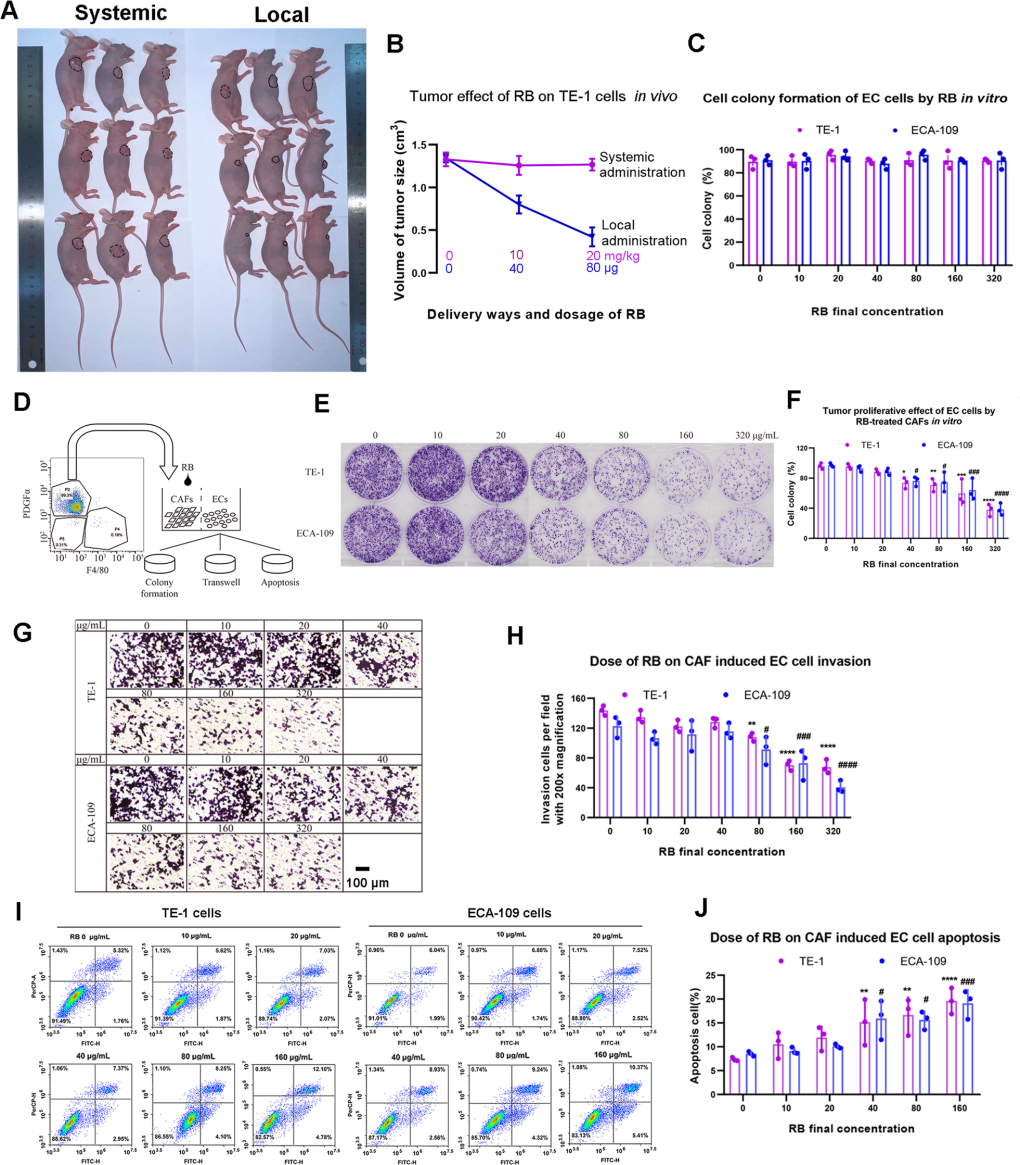
The effect of RB on EC cells in vivo and in vitro*.* **A**, **B** Xenograft nude mice with EC TE-1 cells treated by locally or systemically different dosages of RB (**A**). Tumor size of xenograft EC TE-1 cells by local (blue) and systemic (red) administration of RB (**B**). **C** Tumor proliferation ability of TE-1 and ECA-109 cells by EC cells direct-treated with different concentrations of RB in vitro using colony formation assay. **D** The schematic diagram of isolation of nude mice’s CAFs and cell co-culture system. **E**, **F** Tumor proliferation ability of TE-1 and ECA-109 cells by CAFs treated with different concentrations of RB in vitro using colony formation assay. **G**, **H** Tumor invasion ability of TE-1 and ECA-109 cells by CAFs treated with different concentrations of RB in vitro using transwell assay. **I**, **J** Tumor apoptosis of TE-1 and ECA-109 cells by CAFs treated with different concentrations of RB in vitro using flow cytometry assay. Data is showed as the mean ± standard deviation for at least three independent duplications. Analysis of multiple groups comparison were performed using one-way or two-way ANOVA followed by Dunnett's multiple comparisons test compared with control. The *p*-value less than 0.05 was considered as statistical significance. ^*,#^*P* < 0.05, ^**,##^*P* < 0.01,^***,###^*P* < 0.001 compared with each control

Initially, the common used narcotic drug RB was used to examine the effect on EC. Xenograft mice with EC TE-1 cells were locally (0, 40 and 80 μg per mouse) and systemically (0, 10 and 20 mg/kg) administrated with different doses of RB. We observed that the size of tumor was significantly reduced starting from 40 μg RB (local administration) in TE-1 but not by systemic administration (Fig. 1A, B). Although local administration of RB presented inhibition on EC tumor progress in vivo, we failed to find any significant effect on EC cell proliferation with the direct stimulation of RB in vitro (Fig. 1C, Supplementary data 1).

We suspected that RB might play an indirect role in the inhibitory effect on EC cells by altering TME. Therefore, we isolated the murine PDGFRα^+^ and F4/80^−^ CAFs at the border of tumor, and co-cultured with EC cells with RB treatment (Fig. 1D). Interestingly, we noticed that RB could cause obvious tumor suppression on EC cells after RB was added into fibroblasts for 12 h. RB treatment inhibited cell proliferation and invasion (Fig. 1E–H), and significantly increased cell apoptosis rate (Fig. 1I, J), both of which were dose-dependent.

**Fig. 2:**
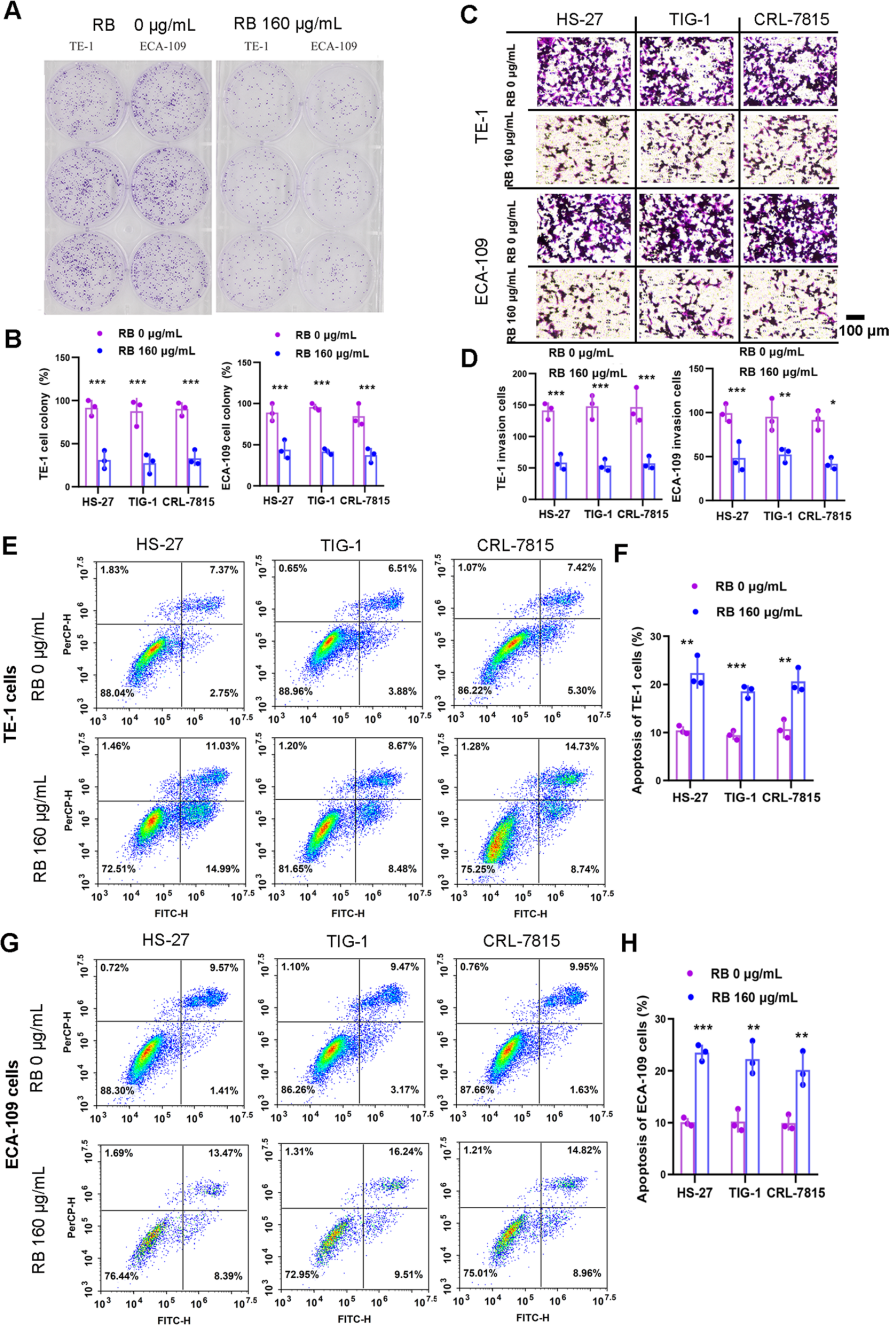
The responses of EC cells by human fibroblasts treated with 160 μg/mL RB in vitro*.* **A**, **B** Tumor proliferation ability of TE-1 and ECA-109 cells by human fibroblasts HS-27, TIG-1 and CRL-7815 treated with 160 μg/mL RB in vitro using colony formation assay. **C**, **D** Tumor invasion ability of TE-1 and ECA-109 cells by human fibroblasts HS-27, TIG-1 and CRL-7815 treated with 160 μg/mL RB in vitro using transwell assay. **E**, **F** Tumor apoptosis of TE-1 cells by human fibroblasts HS-27, TIG-1 and CRL-7815 treated with 160 μg/mL RB in vitro using flow cytometry assay. **G**, **H** Tumor apoptosis of ECA-109 cells by human fibroblasts HS-27, TIG-1 and CRL-7815 treated with 160 μg/mL RB in vitro using flow cytometry assay. All experiments are performed three times at least, and data are presented as the mean ± standard error. ^*^P < 0.05, ^**^P < 0.01, ^***^P < 0.001

Due to the absence of commercial esophageal fibroblasts, we tested the effects of multiple human fibroblasts derived from different tissues (skin HS-27; lung TIG-1; dermal CRL-7815) by 160 μg/mL RB. We noticed that the proliferation abilities of EC cells, both TE-1 and ECA-109, were inhibited (Fig. 2A, B), the invasive abilities were reduced (Fig. 2C, D) and the apoptosis percentages were elevated (Fig. 2E–H) by 160 μg/mL RB with significant statistical differences compared to control. Taken together, we determined that RB could indeed suppress the malignancy of EC cells by affecting the adjacent fibroblasts (see Additional file 1: Fig. S1).

### The transcriptomic changes of fibroblasts induced by RB

**Fig. 3:**
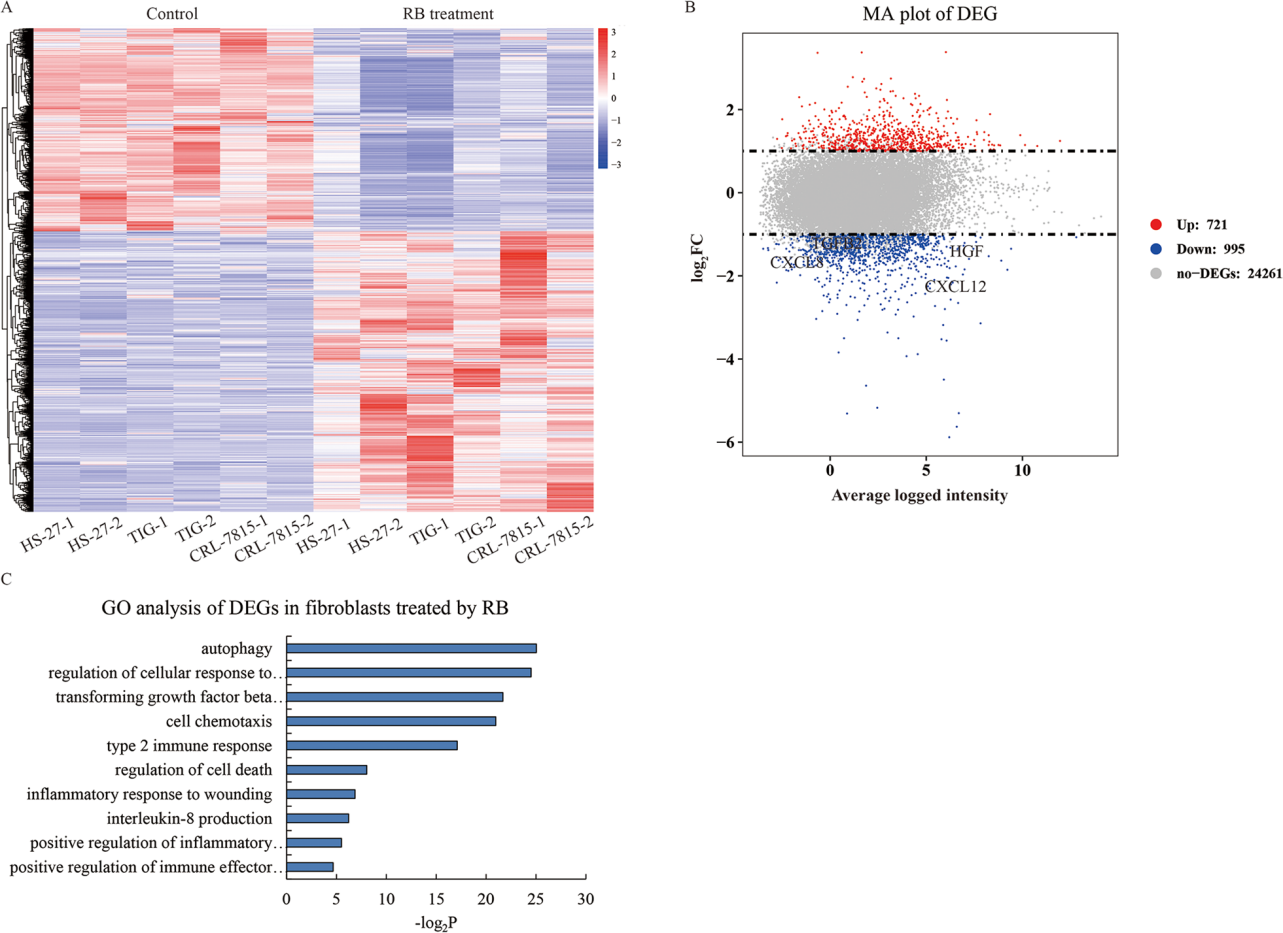
RNA profiles of human fibroblasts affected by RB. **A** Heatmap shows the DEGs (log_2_FC > 1 or < − 1, *P* < 0.05) among HS-27, TIG-1 and CRL-7815 cells with and without RB treatment. **B** MA plot shows 721 up-regulated (red) and 995 down-regulated (blue) genes compared between with and without RB treatment. CXCL12 is highlighted (log_2_FC = − 2.218, *P* < 0.001). **C** Bar charts shows GO and KEGG analysis of the differential expressed genes

To restore the true effects of CAFs by RB in vivo, we studied the global transcriptomic signatures of human fibroblasts to find the target genes that respond to RB (Fig. 3A). Compared with the control group, the intersection of differential expression gene (DEGs) among HS-27, TIG-1 and CRL-7815 cells treated with RB determined 721 up-regulated genes and 995 down-regulated genes (Fig. 3B, Additional file 2: Table S1). Due to the indirect effect of RB on EC cells through CAFs, we mainly focused on the exocrine proteins from fibroblasts. Here, we noticed that a large number of cytokines such as HGF (log_2_FC = − 1.87, *P* = 8 × 10^–14^), TGF-β2 (log_2_FC = − 1.38, *P* = 4.26 × 10^–6^), CXCL12 (log_2_FC = − 2.218, *P* = 7.87 × 10^–4^) and CXCL8 (IL8) (log_2_FC = − 1.85, *P* = 0.011) were down-regulated by RB. Gene Ontology (GO) analysis showed that RB impacted the cellular functions on autophagy, immune and inflammatory response, chemotaxis and cell death, which were closely related to the above cytokines (Fig. 3C). Taken together, we preliminary determined that multiple cytokines secreted by fibroblasts to microenvironment may contribute to the malignant progression of EC.

### Reduced CXCL12 contributed to EC suppression

**Fig. 4:**
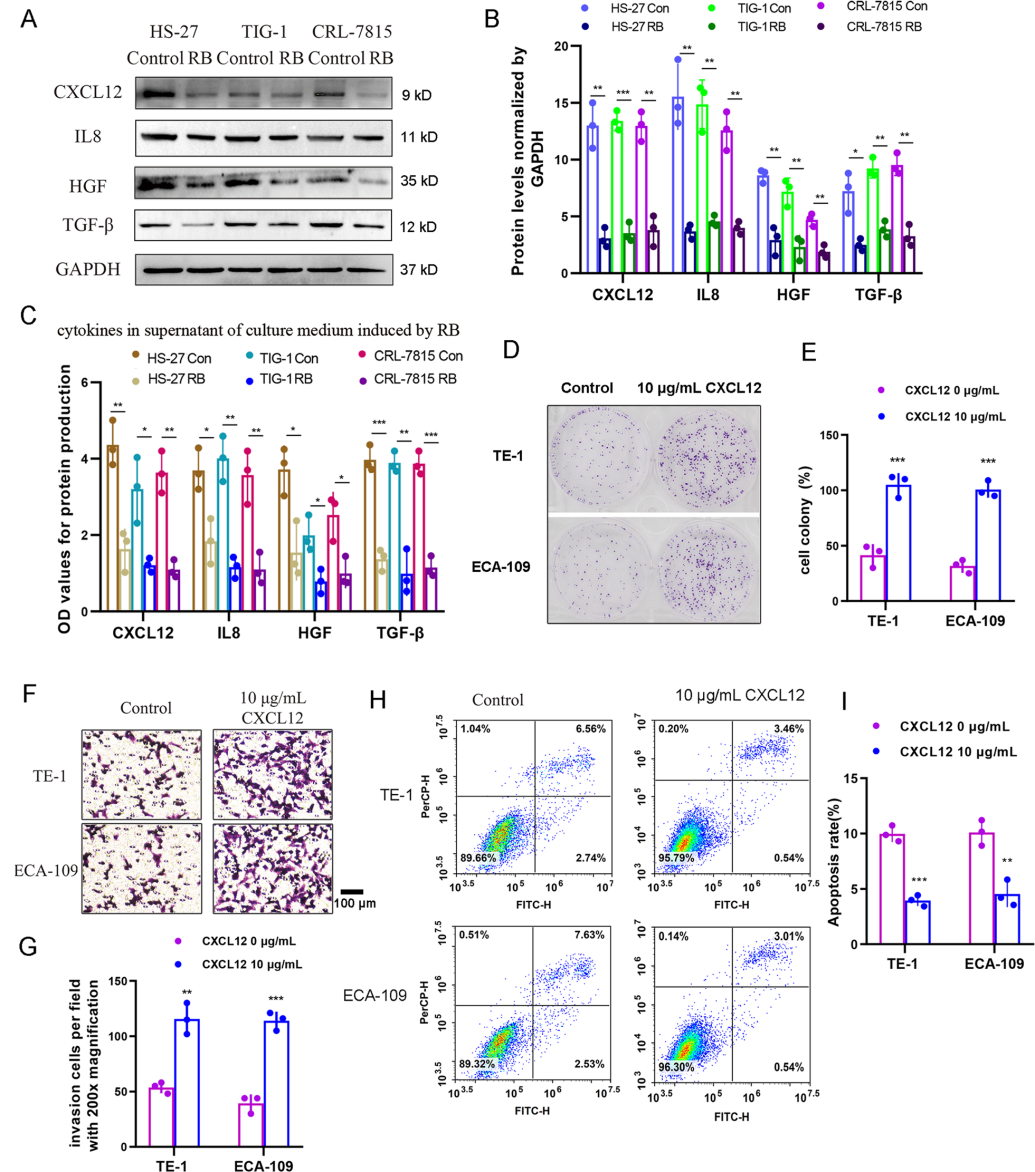
The tumor suppressive effect of CXCL12 on EC malignancy. **A** The expression of cytokines including CXCL12, IL8, HGF and TGF-βin human fibroblasts HS-27, TIG-1 and CRL-7815 induced by RB using WB assay. **B** The statistical analysis of WB assay. **C** The expression of cytokines including CXCL12, IL8, HGF and TGF-βin culture medium supernatant of human fibroblasts HS-27, TIG-1 and CRL-7815 induced by RB using ELISA assay. **D**, **E** Tumor proliferation ability of TE-1 and ECA-109 cells treated with 10 μg/mL CXCL12 using colony formation assay. **F**, **G** Tumor invasion ability of TE-1 and ECA-109 cells treated with 10 μg/mL CXCL12 using transwell assay. **H**, **I** Tumor apoptosis of TE-1 and ECA-109 cells treated with 10 μg/mL CXCL12 using flow cytometry assay. Data are presented as the mean ± standard error, n ≥ 3. ^*^P < 0.05, ^**^P < 0.01, ^***^P < 0.001

Next, WB assay was used to detect CXCL12, IL8, HGF and TGF-β in HS-27, TIG-1 and CRL-7815 cells (Fig. 4A, B). We confirmed that these cytokines were all significantly reduced by RB treatment compared to the untreated control (CXCL12: *P* < 0.01 in HS-27 and CRL-7815, *P* < 0.05 in TIG-1; IL8: *P* < 0.05 in HS-27, *P* < 0.01 in TIG-1 and CRL-7815; HGF: *P* < 0.05 in HS-27, TIG-1 and CRL-7815; TGF-β: *P* < 0.001 in HS-27 and CRL-7815, *P* < 0.01 in TIG-1). Consistently, the compromised cytokines CXCL12, IL8, HGF and TGF-β within the supernatant of culture medium were also displayed by ELISA (Fig. 4C). Here we only focused on CXCL12. The direct compensation of 10 μg/mL CXCL12 recombinant protein into EC cells without fibroblast co-culture could substantially promote the malignancy of ECA-109 and TE-1 cells, including cell proliferation (TE-1: *P* = 0.001; ECA-109: *P* < 0.001, Fig. 4D, E), invasion (TE-1: *P* < 0.01; ECA-109: *P* < 0.001, Fig. 4F, G) and apoptosis (TE-1: *P* = 0.001; ECA-109: *P* < 0.01, Fig. 4H, I). Taken together, we concluded that reduction of CXCL12 contributed to tumor suppression of EC cells in *vitro.*

### CXCL12 regulated by RB-mediated PI3K/AKT/mTOR pathway in fibroblasts

**Fig. 5:**
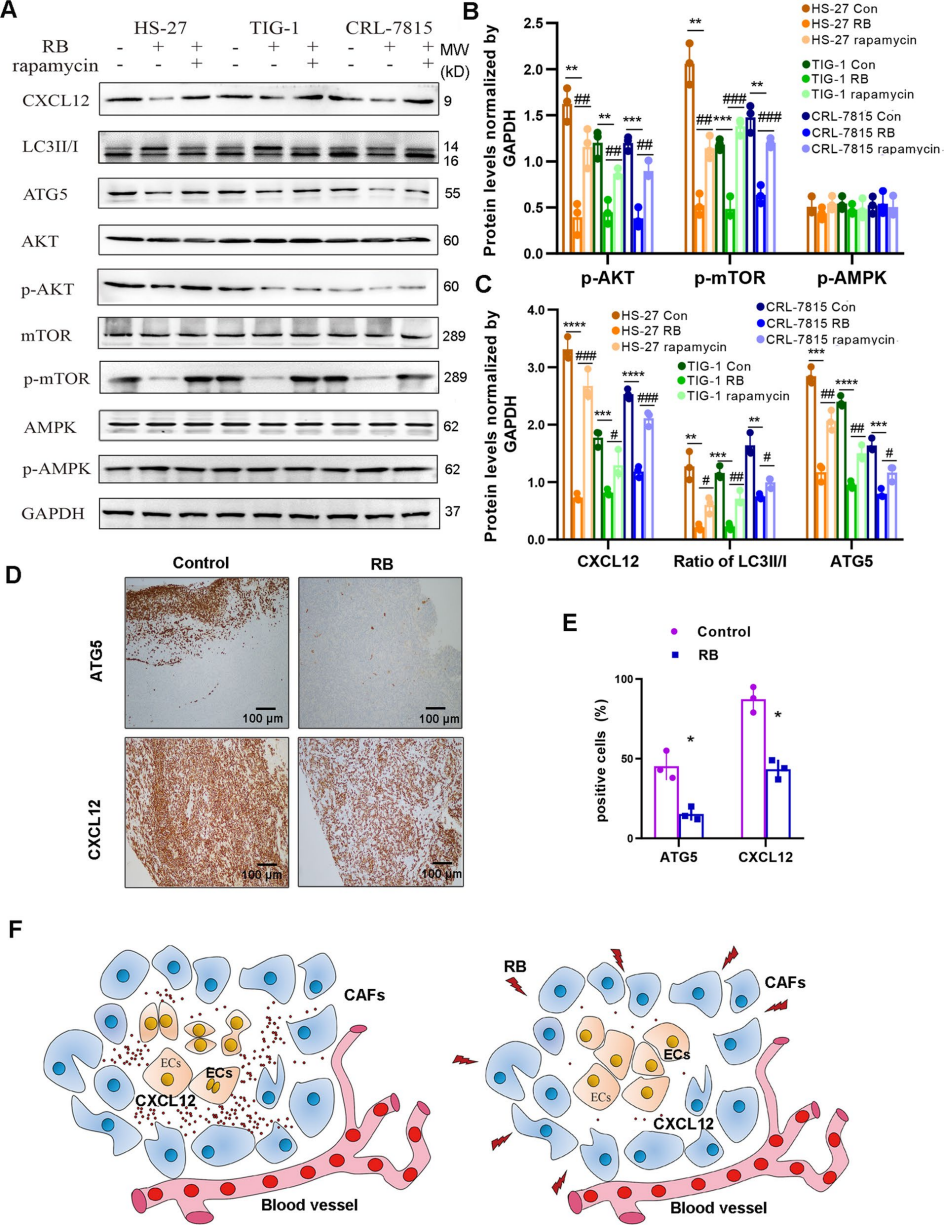
Activation of autophagy in fibroblasts induced by RB. **A** The activities of PI3K/AKT/mTOR, and the expression of LC3II/I, ATG5 and CXCL12 in human fibroblasts HS-27, TIG-1 and CRL-7815 induced by RB using WB assay. **B** The statistical analysis of the phosphorylated kinases of WB assay. **C** The statistical analysis of the expression of LC3II/I, ATG5 and CXCL12. **D** The expression of ATG5 and CXCL12 in CAFs of xenograft mice in vivo using IHC assay. Images are shown with 100 magnification. **E** The statistical analysis of ATG5 and CXCL12 of IHC assay. **F** Graphical overview of this study. RB plays a tumor suppressive role in EC through governing the CXCL12 secretion from CAFs. Data are presented as the mean ± standard error, n ≥ 3. ^*, #^*P* < 0.05, ^**, ##^*P* < 0.01, ^***, ###^*P* < 0.001 compared with each control

Finally, we detected PI3K/AKT/mTOR signaling pathway associated with CXCL12 in RNA-seq (Fig. 3C). Again, HS-27, TIG-1 and CRL-7815 cells were added RB and rapamycin to study the effect of autophagy on CXCL12 expression in fibroblasts. We observed that the phosphorylation levels of AKT and mTOR were indeed diminished by RB, but reversed by addition of rapamycin, whereas the phosphorylation of AMPK did not change (Fig. 5A, B). Consistently, the expression levels of LC3-II/I, ATG5 and CXCL12 were all reduced by RB, and rescued by rapamycin (Fig. 5A, C), indicating that the activity of autophagy was blocked by RB through PI3K/AKT pathway in fibroblasts. Furthermore, we investigated the activity of autophagy in CAFs of xenograft mice in vivo. IHC assay showed that ATG5 and CXCL12 were both remarkably down-regulated in RB treated mice (Fig. 5D, E). Overall, we summarized that RB could repress autophagy to block the CXCL12 secretion in CAFs and weaken the CXCL12-mediated tumor promotion of EC (Fig. 5F).

## Discussion

In the present study, we demonstrate that RB exerts suppressive effect on EC progression through governing the CXCL12 secretion from CAFs. RB could repress PI3K/AKT/mTOR signaling pathway and autophagy to block the CXCL12 secretion from CAFs. Accumulating evidence from preclinical studies has determined that adrenergic-inflammatory pathways contributing to tumor malignancy can be modulated by anesthetics [29]. General anesthesia including intravenous and inhalational ways are implicated with long-term cancer outcomes. However, general anesthesia is reported to be associated with tumor progression [30–32]. On the contrary, two in vivo animals with regional anesthesia show beneficial effect [33, 34]. Due to the lack of underlying molecular and cellular mechanisms so far, the role of regional anesthesia in cancer remains unclear.

We chose the commonly used narcotic drug RB to examine its effect on EC in vivo and in vitro. And found RB suppressed the malignancy of EC cells by affecting the adjacent fibroblasts. In our in vivo study, systemic administration of RB did not influence EC growth. In addition to the different metabolic pathways of RB and sevoflurane/propofol, the concentration and duration of RB and the different responses from human and mice all indicate that the complicated and comprehensive synergistic effect of general anesthesia is still hard to figure out [35]. However, the local administration of RB with high-frequency in vivo can indeed exhibit an apparent tumor-repression of EC. Our data suggests that the high local doses of RB may be required to inhibit cancer progression. Therefore, the guideline of anesthesia usage in perioperative practice specific for tumor alleviation needs to be defined and designed. Through isolating the murine PDGFRα^+^ and F4/80^−^ CAFs at the border of tumor, and co-culturing with EC cells with RB treatment, we found RB could cause obvious tumor suppression on EC cell malignant phenotype, suggesting that RB could indeed suppress the malignancy of EC cells by affecting the adjacent fibroblasts.

Furthermore, our study reveals that human fibroblast cell lines derived from different tissue sources can secrete CXCL12 and other cytokines, and the expression of these cytokines can be down-regulated by RB, indicating a conservative effect on regulating CXCL12 in fibroblasts by RB. It also suggests that the role of RB in CAFs is universal in various tissue-specific cancers. Our data further determines that CXCL12 is up-regulated through the activation of PI3K/AKT/mTOR-mediated autophagy. Nevertheless, our in vitro study presents that this regulatory signaling pathway in EC cells is seemingly not affected by RB. Autophagy plays a key role in the pathogenesis and outcome of EC patients [36]. Autophagy related prognostic gene markers and autophagy regulatory factors have attracted the attention of researchers in recent years [37–40]. The change of LC3-II/I ratio is usually used to evaluate autophagy formation. During the formation of autophagy, cytoplasmic LC3 will enzymatically hydrolyze a small segment of polypeptide to form LC3-I, and LC3-I will combine with PE to convert into autophagy membrane type LC3-II. The increase of LC3-II/I ratio indicates that autophagy level is up-regulated. In this study, fibroblasts have autophagy to a certain extent, which is beneficial to maintain their normal biological functions. RB reduces the ratio of LC3-II/I and also reduces the expression of CXCL12, which can be blocked by the autophagy inducer Rapamycin, indicating that RB inhibits the autophagy pathway of fibroblasts and the expression of CXCL12, thus promoting the malignant progression of EC.

We studied the global transcriptomic signatures of human fibroblasts to find the target genes responding to RB. From our RNA-seq data, we have unmasked a large number of biomarkers. Our future studies plan to focus on these target biomarkers which may play a particular role in responding to RB in fibroblasts. Our findings provide a novel insight into the underlying mechanism on EC suppression by RB, emphasizing the importance of tumor microenvironment in modulating cancer progress and development, and suggest new potential therapeutic thoughts in EC treatment. Nevertheless, the regulatory mechanism of RB needs to be further explored, such as how autophagy of fibroblasts regulates the expression of CXCL12. In addition, RB injection will cause severe local pain [41]. Considering the dose limit of RB, it is of great research value to combine low dose RB with anti-tumor chemotherapy drugs to improve the sensitivity and therapeutic effect of chemotherapy drugs.

## Conclusions

Our data suggest that RB could repress PI3K/AKT/mTOR signaling pathway and autophagy to block the CXCL12 expression in CAFs, thereby weakening the CXCL12-mediated EC tumor progression. Our data provide a novel insight into the underlying mechanism of RB inhibiting EC, and emphasize the importance of tumor microenvironment (cytokines from CAFs) in modulating cancer malignant progression. In the future, we will further study the anti-tumor effect and mechanism of RB, and observe the anti-tumor effect of RB combined with other drugs.

## Supplementary Information

**Additional file 1: Figure S1.** Cell proliferation ability of EC TE-1 and ECA-109 cells treated by different dosages of RB via colony formation assay

**Additional file 2: Table S1.** DEGs of human fibroblasts treated by RB. DEGs screening condition is (log_2_FC > 1 or < -1, *P* < 0.05)

## Data Availability

RNA-sequencing data was uploaded to ArrayExpress (E-MTAB-11728).
