# Supplementary figures and images for: Rocuronium bromide suppresses esophageal cancer via blocking the secretion of C–X–C motif chemokine ligand 12 from cancer associated fibroblasts
Source: J Transl Med. 2023 Apr 8;21:248. doi: 10.1186/s12967-023-04081-y (PMC10082495; doi:10.1186/s12967-023-04081-y)

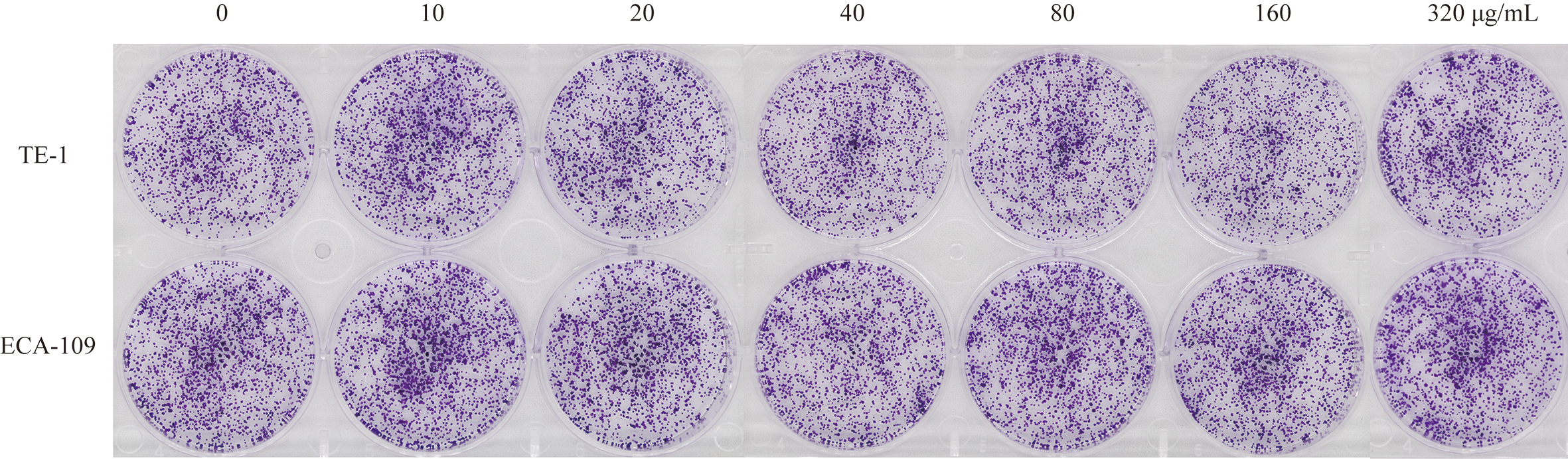

Supplement: Supplementary file 1 — Additional file 1: Figure S1. Cell proliferation ability of EC TE-1 and ECA-109 cells treated by different dosages of RB via colony formation assay [file 12967_2023_4081_MOESM1_ESM.tif]
